# Presumed Exposure to Chemical Pollutants and Experienced Health Impacts among Warehouse Workers at Logistics Companies: A Cross-Sectional Survey

**DOI:** 10.3390/ijerph18137052

**Published:** 2021-07-01

**Authors:** Szabolcs Lovas, Károly Nagy, János Sándor, Balázs Ádám

**Affiliations:** 1 Department of Public Health and Epidemiology, Faculty of Medicine, University of Debrecen, 26 Kassai Street, 4028 Debrecen, Hungary; lovas.szabolcs@med.unideb.hu (S.L.); nagy.karoly@med.unideb.hu (K.N.); sandor.janos@med.unideb.hu (J.S.); 2 Doctoral School of Health Sciences, University of Debrecen, 4032 Debrecen, Hungary; 3 Institute of Public Health, College of Medicine and Health Sciences, United Arab Emirates University, Al Ain P.O. Box 17666, United Arab Emirates

**Keywords:** chemical pollutant, freight container, logistics, pesticide, volatile organic compound, warehouse

## Abstract

During intercontinental shipping, freight containers and other closed transport devices are applied. These closed spaces can be polluted with various harmful chemicals that may accumulate in poorly ventilated environments. The major pollutants are residues of pesticides used for fumigation as well as volatile organic compounds (VOCs) released from the goods. While handling cargos at logistics companies, workers can be exposed to these pollutants, frequently without adequate occupational health and safety precautions. A cross-sectional questionnaire survey was conducted among potentially exposed warehouse workers and office workers as controls at Hungarian logistics companies (1) to investigate the health effects of chemical pollutants occurring in closed spaces of transportation and storage and (2) to collect information about the knowledge of and attitude toward workplace chemical exposures as well as the occupational health and safety precautions applied. Pre-existing medical conditions did not show any significant difference between the working groups. Numbness or heaviness in the arms and legs (AOR = 3.99; 95% CI = 1.72–9.26) and dry cough (AOR = 2.32; 95% CI = 1.09–4.93) were significantly associated with working in closed environments of transportation and storage, while forgetfulness (AOR = 0.40; 95% CI = 0.18–0.87), sleep disturbances (AOR = 0.36; 95% CI = 0.17–0.78), and tiredness after waking up (AOR = 0.40; 95% CI = 0.20–0.79) were significantly associated with employment in office. Warehouse workers who completed specific workplace health and safety training had more detailed knowledge related to this workplace chemical issue (AOR = 8.18; 95% CI = 3.47–19.27), and they were significantly more likely to use certain preventive measures. Warehouse workers involved in handling cargos at logistics companies may be exposed to different chemical pollutants, and the related health risks remain unknown if the presence of these chemicals is not recognized. Applied occupational health and safety measures at logistics companies are not adequate enough to manage this chemical safety issue, which warrants awareness raising and the introduction of effective preventive strategies to protect workers’ health at logistics companies.

## 1. Introduction

Logistics is one of the world’s largest economic sectors with a diverse scope of work activities that involve a significant proportion of the labor force. The transportation and storage of goods and packaging materials are the two major objectives of logistics, involving the complex process from collecting newly manufactured products from producers through their warehousing at retail locations and distribution to the costumers. It refers to the overall process of managing how goods are acquired, stored, and transported to their final destinations, often to another continent. The major goal of logistics is to meet consumer requirements in a well-timed, cost-effective manner through specialized logistics companies worldwide. Intercontinental transportation is mainly carried out by container ships and cargo flights all over the world; however, freight transport by sea is the most cost-effective way to connect different continents. In Europe, the inland transport of goods is mainly accomplished by trucks and freight trains due to the relatively small distances between European countries. For the transportation of freights, the preferred way is using containers and closed cargo bays to deliver shipments from the producers to the consumers, ensuring protected environments for the goods [1].

Trans-continental shipping and inland transportation require closed and relatively dry conditions with almost zero aeration in many cases, since the goods have to be protected from humidity and other environmental factors that could deteriorate their quality. Closed spaces of transportation and storage are safe and applicable to carry out international and midland transportation and warehousing of goods, but they can be polluted with several chemicals. Pollutants accumulating in the air can expose employees, who handle cargo in these closed environments, predominantly by inhalation, consequently exposing them to health risks [2,3,4].

Goods can spend quite a long time in closed transport devices and warehouses; meanwhile, volatile organic compounds (VOCs) and pesticide residues can accumulate, expose workers without appropriate preventive measures, and induce various acute and chronic health effects [5,6,7].

Under the regulation of the United Nations International Plant Protection Convention (IPPC), transporting wood materials of a thickness greater than 6 mm, which are used to ship products between countries, have to be treated to prevent the spread of harmful organisms that could negatively affect plants or ecosystems [8]. For this purpose, either fumigation or heat treatment is used. The procedure of heat treatment, which requires a minimum period of 30 min at a constant temperature of 56 °C, is complicated and expensive, which presumably makes chemical treatment favorable in practice. Methyl bromide used to be the most frequently applied fumigant that has recently been phased out in conjunction with the Montreal Protocol on Substances that Deplete the Ozone Layer, as it turned out to be an ozone-depleting substance [9]. However, its usage is still allowed in case of critical use exemptions when an adequate alternative is not available [10,11]. To substitute methyl bromide, several other chemicals are used for fumigation nowadays, such as sulfuryl fluoride, phosphine, and chloropicrin. The peak concentrations of fumigant residues in closed spaces can reach as high as 70% of their administered concentrations, which warrants the use of well-defined obligatory safety precautions during the handling of fumigated transport containers and closed cargo bays [3,5].

In addition to the undesirable exposure caused by the residues of fumigants and other pesticides, there are several VOCs released from transported goods and packaging materials, which contribute to the complexity of chemical exposures in transportation and trade [12]. The most commonly released VOCs in transport containers are benzene, xylene, ethyl benzene, toluene, chloromethane, and formaldehyde. These chemicals are emitted from goods, such as furniture, electrical appliances, shoes, and textiles that have been manufactured with the use of paint, lacquer, or glue [2,3,12]. According to a study carried out in Hamburg, Germany, 70% of transport containers arriving in the harbor were contaminated with toxic chemicals above chronic reference exposure levels, and 36% of them exceeded the higher acute reference exposure level thresholds. The chemicals were fumigants and VOCs, such as benzene, formaldehyde, ethylene oxide, hydrogen phosphide, and methyl bromide [7].

Exposure to carbon monoxide can also pose an occupational hazard in closed transport devices and warehouses when proper ventilation is not in place to ensure harmless indoor air quality. Newly manufactured wood pellets are frequently used in the transportation and storage of goods. The emission of VOCs and carbon monoxide from wood pellets is a common problem at pellets producers where high levels of hexanal and carbon monoxide can be detected, constituting an occupational health hazard. Although such exposure occurs mainly from newly manufactured wood pellets in warehouses, these chemicals can be released during the transportation and storage of goods, too, leading to continuous exposure inside closed spaces [10,13].

Another widespread chemical exposure in transportation and storage is diesel exhaust emitted by trucks and forklifts during handling freights. Although the largest proportion of diesel exhaust quits to the open air, the working environment can be polluted with its harmful components [14,15].

Considering the various potential sources of chemical pollutants, workers employed in transportation and storage can be exposed to several noxious substances, even without recognizing the risk [4,5,6,12,16]. The aim of this study was to investigate presumed occupational chemical exposures and their experienced health impacts, as well as the related knowledge, attitudes, and practices through a cross-sectional questionnaire survey among workers at Hungarian logistics companies.

## 2. Materials and Methods

### 2.1. Study Design

A cross-sectional questionnaire survey was carried out among workers employed at Hungarian logistics companies to investigate their exposure to chemical pollutants occurring in closed spaces of transportation and storage, collecting information about experienced health effects, knowledge of and attitude to workplace chemical exposures, as well as about the occupational health and safety precautions applied.

The sampling consisted of two steps. First, logistics companies handling at least partly non-dangerous goods, located in Hungary, and being a member of the Association of Hungarian Logistic Service Centers were invited to participate in the study. In Hungary, the majority of the logistics companies have membership in this association, which provided a member list, including all registered logistics companies handling non-dangerous goods, along with a letter supporting the study. All the 35 registered logistics companies were contacted, from which seven companies agreed to participate in the study.

The enrolled logistics companies perform the transportation and storage of everyday products (e.g., electrical equipment, industrial products, disinfectants, clothes, foods and consumable goods, etc.).

### 2.2. Questionnaire Design and Data Collection

The survey questionnaire was developed based on a previously applied questionnaire in Australia [17], which was supplemented with standardized question blocks from the Tobacco Questions for Survey (WHO) [18], the AUDIT–C alcohol consumption survey [19], and the European Health Interview Survey (EHIS) [20]. Since the original questionnaire focused on chemical hazards in shipping containers only, our questionnaire was modified to be applicable to handling cargos in warehouses, too. The questionnaire was adapted to the Hungarian context and translated into Hungarian by one author (SzL) and back into English by another author (KN), independently. The original and the back-translated English versions were compared, and the Hungarian version was revised by the senior researcher (BÁ) in order to refine the clarity of the questions.

The questionnaire consisted of five parts: (1) background information and lifestyle factors, (2) occupational and exposure history, (3) diagnosed health problems, (4) symptoms that can be related to chemical exposures at work, and (5) workplace chemical exposures and applied preventive measures. The fifth part of the questionnaire was completed only by the warehouse workers, as this section only covered questions on working in warehouses and closed transport devices.

The questionnaire collected information about the participants’ age, gender, lifestyle factors (e.g., smoking status and alcohol consumption), occupational history, medical conditions, and potentially work-related symptoms. The questionnaire also obtained data on activities of warehouse and office work, past and present work with chemicals, as well as the warehouse workers’ knowledge, attitudes, and preventive practices regarding chemical pollutants in closed spaces of transportation and storage.

A pilot test was carried out at a Hungarian logistics company with the participation of six office workers and six warehouse workers. The questionnaire was finalized based on the feedback from the participants.

Questionnaires were distributed among all warehouse and office workers of the participating companies located in various Hungarian cities with the help of warehouse managers. The inclusion criteria were (1) adults aged between 18 and 65; (2) the place of daily work is the logistics company where questionnaires were distributed; (3) in case of office workers, no working activity in closed spaces of warehouses; (4) in case of warehouse workers, working activity in closed spaces of warehouses; and (5) agreement to fill in the questionnaire. The contacted working population constituted of 165 warehouse and 185 office workers. The participants formed two comparative groups: employees working inside warehouses and closed transport devices (e.g., transport containers) and workers in office settings at the logistics companies, being not involved directly in handling goods.

A brief introduction about the aim of the study and about the questionnaire was presented to the participants by a researcher, when they were also able to ask questions. After giving written consent for participation, warehouse and office workers completed the questionnaire independently.

### 2.3. Data Analysis

Collected data were managed and descriptively analyzed in Microsoft^®^ Excel 2016. Inferential statistical analysis was carried out in STATA^®^ version 12.0. The main exposure variable was occupational exposure status determined by job category (handling goods in closed spaces of transportation and storage vs. working in office settings). For the assessment of knowledge, attitude, and preventive measures, the main independent variable was the participation in specific workplace health and safety training about chemical pollutants.

In univariate analysis, an independent *t*-test was used for continuous variables and a chi-square test was used for ordinal and categorical variables to determine significant predictors. A multivariate logistic regression model was constructed to adjust for potential confounders. Significance was accepted at *p* < 0.05.

## 3. Results

A total of 258 workers employed at seven Hungarian logistics companies participated in the survey, including 122 warehouse workers who were considered to be potentially exposed to chemicals at work, and 136 office workers functioning as controls who were occupationally not exposed to chemicals. The response rate was 73.5% among office and 73.9% among warehouse workers.

### 3.1. Sociodemographic Characteristics, Lifestyle, and Chemical Exposure History

The majority of the warehouse workers were males (92.6%), while the control group consisted mainly of females (79.4%). The mean age of the employees working in closed spaces of transportation and storage was not significantly different from the controls (41.2 ± 0.93 vs. 38.1 ± 0.89 years).

The frequency of smoking (daily or less than daily) was not significantly different among warehouse workers compared to the controls (48.4% vs. 41.2%), similarly to workplace secondhand smoke exposure (32.8% vs. 16.2%); however, alcohol consumption was significantly higher among warehouse workers (proportion of monthly or more often 93.4% vs. 83.8%).

Only 9.1% of the warehouse workers indicated recent or past occupational exposure to chemicals, while none of the office workers did, but medication use was not significantly different between the groups (39.3% vs. 47.8%). Table 1 displays the sociodemographic characteristics, lifestyle, and chemical exposure of the participants.

### 3.2. General Health Status

Severe headache (crude odds ratio (COR) = 0.51; 95% confidence interval (CI) = 0.26–0.99) and chronic depression (COR = 0.19; 95% CI = 0.04–0.87) showed significant association with working in office settings, but the association disappeared after adjustment for potential confounders (Table 2). Other medical conditions did not show any significant difference between the working groups.

### 3.3. Symptoms

Numbness or heaviness in the arms or legs, trembling of hands, slurred speech, and the unpleasant taste in the mouth were reported significantly more frequently among warehouse workers than among controls. After adjustment for potential confounders, numbness or heaviness in the arms or legs (AOR = 3.99; 95% CI = 1.72–9.26) and dry cough (AOR = 2.32; 95% CI = 1.09–4.93) were significantly associated with working in closed spaces of transportation and storage.

Headache, dizziness, nausea, stomach pain or cramps, feeling depressed, rapid changes in mood, forgetfulness, difficulty in concentrating, sleep disturbances, tiredness after wake up, irritation of the eyes, and skin irritation were reported significantly more frequently by office workers. After adjustment for potential confounders, forgetfulness (AOR = 0.40; 95% CI = 0.18–0.87), sleep disturbances (AOR = 0.36; 95% CI = 0.17–0.78), and tiredness after wake up (AOR = 0.40; 95% CI = 0.20–0.79) remained significantly associated with working in office settings. Table 3 shows the frequency of potentially work-related symptoms among warehouse and office workers.

### 3.4. Knowledge of and Attitude to Workplace Chemical Exposures

Of the 122 warehouse workers, 79.5% worked in warehouses or closed transport devices (e.g., transport containers) on a daily basis, while only 1.6% worked rarely (Table 4). The majority of them (62.3%) have never heard about chemical pollutants in their workplace; for those who heard, workplace health and safety training was the most typical source. Most (65.6%) warehouse workers indicated lack of knowledge about this workplace issue, while only 9.8% of the respondents reported comprehensive knowledge related to the chemical pollutants. Less than half (47.5%) of the warehouse workers thought that they never work in polluted occupational environments, while only 5.7% estimated the frequency of this working conditions to be often and 12.3% always. The majority of them (39.3%) estimated that being exposed by the pollutants is extremely unlikely, while only 8.2% estimated this scenario to be likely and 9% estimated this scenario to be extremely likely. Concerning the harmfulness of the pollutants, 21.3% of the workers believed the pollutants to be not harmful, 27.1% considered them to be moderately harmful, while 8.2% estimated the pollutants to be extremely harmful.

A total of 38 warehouse workers (31.1%) have completed specific occupational health and safety training, where they were informed, among others, about potential chemical exposures in the workplace, while 68.9% have never participated in such trainings (Table 5). For those who have completed such training, the most frequently covered topics were the selection and proper use of personal protective equipment (89.5%) and the way of reporting incidents of contamination (81.6%). Administrative control measures to prevent chemical exposure (47.4%) and properties of chemical pollutants that may help to identify exposure (36.8%) were reported to be the least covered topics in the trainings.

Association between completing specific occupational health and safety training related to chemical pollutants in closed spaces of transportation and storage and the knowledge of and attitude to workplace chemical exposures were detected among warehouse workers. Workers who accomplished such training were more aware of (AOR = 7.85; 95% CI = 3.20–19.27), and had more detailed knowledge about the pollutants compared to those who did not participate in such training (AOR = 8.18; 95% CI = 3.47–19.27). They thought more frequently working in closed spaces of transportation and storage that can likely be polluted with chemicals (AOR = 2.67; 95% CI = 1.24–5.78), creating a harmful work environment (AOR = 2.21; 95% CI = 1.06–4.63) (Table 6).

### 3.5. Workplace Preventive Measures

The most frequently applied occupational health and safety precautions reported by the warehouse workers were wearing of personal protective equipment (66.4%) followed by the use of natural ventilation to remove the pollutants from the air of closed spaces (31.9%) (Table 7). Other reported preventive measures were taking reasonable care when opening transport containers and closed transport devices to avoid exposure (23.8%), checking documentation of transport devices that may emit chemical fumes (22.1%), extracting the pollutants using mechanical ventilation (10.7%), and, least frequently, monitoring the air quality inside warehouses and closed spaces of transportation (4.9%). Twenty-three of the 122 warehouse workers (18.6%) answered that they do not take any specific precautions to prevent exposure to chemical pollutants.

Warehouse workers who completed specific occupational health and safety training related to chemical pollutants were more likely to apply certain preventive measures in practice (Table 8). Checking the documentation of potentially polluted transport devices (AOR = 5.49; 95% CI = 2.13–14.14), opening transport devices taking reasonable care to avoid the exposure to pollutants (AOR = 7.16; 95% CI = 2.78–18.44), using the blower or extractor to remove chemical pollutants (AOR = 8.90; 95% CI = 1.96–40.27) and using natural ventilation to remove pollutants (AOR = 2.48; 95% CI = 1.03–5.96) were significantly associated with the participation in specific training.

Being aware of chemical pollutants in closed spaces of transportation and storage, regardless of the source of information, was also significantly associated with checking the documentation of potentially polluted transport devices (AOR = 2.96; 95% CI = 1.18–7.44) and opening transport devices with reasonable care (AOR = 3.04; 95% CI = 1.25–7.39) (Table 8).

Among workers who participated in specific workplace health and safety training related to chemical pollutants in closed spaces of transportation and storage, the following potentially work-related symptoms were significantly less frequent compared to the workers without such training: dryness of throat and/or mouth (AOR = 0.26; 95% CI = 0.10–0.64), throat irritation (AOR = 0.33; 95% CI = 0.14–0.81), dry cough (AOR = 0.43; 95% CI = 0.19–0.97), and diarrhea (AOR = 0.38; 95% CI = 0.16–0.91) (Table 9).

## 4. Discussion

Our cross-sectional questionnaire survey investigated the experienced health effects of potential occupational chemical exposures among warehouse workers, and their related knowledge, attitudes, and preventive practices. Compared to office workers, no diagnosed medical problems were significantly associated with warehouse work. Although asthma, bronchitis/COPD, and allergy were more frequent among warehouse workers, which are medical conditions that may have an occupational background, no clear association could be identified within the scope of this study. Chemical pollutants that may trigger these medical conditions can be, among others, benzene, toluene, xylene, different pesticide residues, and banned persistent organic pollutants (e.g., polychlorinated biphenyles) [21,22,23,24,25,26,27]. Due to the relatively young study population, chronic health problems can be in the preclinical phase and remain undiagnosed for long, manifesting only in old age when a causal relationship is difficult to establish. Dry cough and numbness or heaviness in the arms or legs could be associated with work in closed spaces of transportation and storage, representing respiratory irritation and peripheral neurotoxic effects, which are symptoms potentially induced by chemical exposures to fumigants and various solvents [28,29,30]. Although no significant correlation was found, trembling of hands, muscle cramps, slurred speech, and unpleasant taste in the mouth were more frequent among warehouse workers. These symptoms can be related to various chemical substances that may occur under these circumstances, such as aromatic organic solvents, volatile residues of fumigants, and other toxic industrial chemicals [7,9,12,31,32,33]. Several pesticides and their residues, for instance various fumigants and insecticides, have neurotoxic effects even in low or moderate concentrations, which may be experienced by exposed warehouse workers [34,35,36,37,38]. Dryness of throat and/or mouth, throat irritation, a runny nose, skin irritation, and wheezing in the chest were also more frequent among warehouse workers. These symptoms can be associated with VOCs and pesticide residues in inappropriately ventilated workplace air, such as in transport containers and warehouses [39,40,41,42]. Forgetfulness, sleep disturbances, and consequent tiredness after waking up were found to be significantly associated with office work. These symptoms are frequently reported by office workers and could be, among others, attributed to workplace stress [43,44,45].

Our study revealed that the majority of the warehouse workers (62.3%) have never heard about chemical pollutants in warehouse settings where non-dangerous goods are handled. For those who were aware about this occupational chemical safety issue, the main source of information was the workplace health and safety training. However, the effectiveness of these trainings seems to be questionable. Most of the respondents had no or very limited knowledge about chemical pollutants, and almost half of them thought that they never work in a contaminated environment. Most of the warehouse workers perceived that the chance of being exposed to chemicals in the workplace was extremely unlikely, and the majority estimated the harmfulness of the pollutants to be of no concern for their health [5,46,47].

On the other hand, warehouse workers who completed specific workplace health and safety training related to chemical pollutants had increased knowledge about workplace chemical exposures and expressed their concerns in association with this problem. Dryness of throat and/or mouth, throat irritation, dry cough, and diarrhea were also significantly less frequent among warehouse workers who completed a specific workplace health and safety training about chemical pollutants. This can be explained by their increased knowledge and preventive attitude, more frequently and adequately used health and safety measures.

The use of personal protective equipment (dust masks and protective gloves), and natural ventilation were the most frequently reported occupational health and safety precautions applied by the warehouse workers. Specific additional preventive measures (e.g., mechanical extract ventilation, specific respiratory protective equipment, and standard operating procedures (SOPs) related to container handling) were only applied at a few logistics companies, typically where container handling was performed.

## 5. Limitations

Our study has its potential limitations. The cross-sectional survey design provides a snapshot about this workplace chemical issue but does not allow for establishing causal relationships, which warrants analytical studies to observe the effects of warehouse pollutants on workers’ health. The study population was limited by the low participation rate of the logistics companies, which may threaten the representativeness. Nevertheless, the portfolio and work activities of the enrolled companies well represented the Hungarian logistics industry according to the professional judgement of the investigators.

It can also be reasonably assumed that the companies willing to take part in the study have above-average occupational health and safety consciousness; therefore, the explored level of knowledge, attitudes, and prevention is rather an overestimation of the industrial average. The sample size was relatively low, which may limit the detection of differences of experienced health effects and diagnosed medical conditions between the two groups. Information gathered by the questionnaire was self-reported, the frequency and level of chemical exposures were not measured quantitatively within the framework of the study, and undiagnosed medical conditions may have remained hidden. Finally, the investigated symptoms and medical conditions are multifactorial; therefore, although several potential risk factors were included and controlled for in the statistical analysis, remaining confounders cannot be entirely excluded.

## 6. Conclusions

The occurrence and long-term health risks of exposure to pesticide residues and volatile organic chemical pollutants in warehouses is underestimated by the workers who are involved in handling non-dangerous goods at logistics companies. The study could detect symptoms potentially related to chemical exposures among warehouse workers; however, the harmful effects of these pollutants in low concentrations may require a long time, the investigation of which needs follow-up studies with a large sample size to explore the association of health problems with chemicals accumulated in such environments. More precise exposure assessment by detecting and quantifying pollutant concentrations is also necessary for detailed risk assessment.

According to the findings, occupational health and safety precautions at several logistics companies are not adequate enough to control chemical exposures, since the risks arising from the contamination with pollutants during transportation and storage are not recognized necessarily, and the possibility of working in polluted environments is underestimated by the workers. Specific health and safety measures exist, although they are not consistently used, when a logistics company operates freight containers transported by sea, but to most Hungarian companies, containers and closed transport devices are delivered by trucks and trains, where the opening and unloading of cargo are not considered dangerous activities.

The effectiveness of applying adequate preventive measures, most importantly specifically tailored training, is well demonstrated by our findings. Therefore, raising awareness and increasing knowledge about this largely hidden occupational problem among health and safety professionals as well as logistics workers would be necessary to assure healthy workplaces in the logistics industry.

## Figures and Tables

**Table 1:** Sociodemographic characteristics, lifestyle, and chemical exposure history of warehouse and office workers at Hungarian logistics companies.

Characteristics	Warehouse Workers (*n* = 122)		Office Workers (*n* = 136)		*p*–Value
Sex					
Men	92.6%		20.6%		<0.001
Women	7.4%		79.4%		
Age	41.2 ± 0.93 years (SD)		38.1 ± 0.89 years (SD)		0.302
Smoking status					
Daily	45.9%		39.7%		0.246
Less than daily	2.5%		1.5%		
Never	51.6%		58.8%		
Exposure to secondhand smoke (multiple choice)					
At home	16.4%		11.0%		0.984
At workplace	32.8%		16.2%		
At public place	13.9%		46.3%		
Not at all	36.9%		36.8%		
Frequency of alcohol consumption					
Never	6.6%		16.2%		<0.001
Monthly	31.9%		44.9%		
2–4 times in a month	39.3%		33.8%		
2–3 times in a week	19.7%		4.4%		
4 or more times in a week	2.5%		0.7%		
Time spent at the company	7.5 ± 0.58 years (SD)		6.7 ± 0.49 years (SD)		0.092
Past and present work with chemicals (multiple choice)					
Currently	6.6%		0%		0.002
In the past	2.5%		0%		
No	93.4%		100%		
Prescription drug used in the past 12 months					
Yes	39.3%		47.8%		0.172
No	60.7%		52.2%		

**Table 2:** Diagnosed medical conditions among warehouse and office workers at logistics companies.

Medical Conditions	Warehouse Workers (*n* = 122)	Office Workers (*n* = 136)	COR (95% CI)	AOR (95% CI)
Asthma	25	27	1.04 (0.57–1.91)	1.33 (0.41–4.27)
Bronchitis or COPD	10	4	2.95 (0.90–9.65)	1.86 (0.11–31.05)
High blood pressure	23	26	0.98 (0.53–1.83)	0.41 (0.15–1.18)
Myocardial infarction	0	0	n.a.	n.a.
Coronary heart disease	0	1	n.a.	n.a.
Stroke	0	0	n.a.	n.a.
Diabetes	7	17	0.43 (0.17–1.07)	0.47 (0.10–2.32)
Allergy	52	54	1.13 (0.69–1.85)	1.58 (0.65–3.85)
Stomach/duodenal ulcer	8	15	0.53 (0.22–1.28)	0.50 (0.12–2.06)
Liver dysfunction	1	0	n.a.	n.a.
Cancer	0	4	n.a.	n.a.
Incontinence	3	0	n.a.	n.a.
Severe headache ^1^	16	31	0.51 (0.26–0.99) *	0.77 (0.24–2.43)
Chronic anxiety ^1^	1	4	n.a.	n.a.
Chronic depression ^1^	2	11	0.19 (0.04–0.87) *	0.18 (0.01–2.47)
Other mental health issue ^1^	1	5	0.22 (0.02–1.88)	1.28 (0.02–70.72)

* Significant association (*p* < 0.05). n.a. = not available due to zero or low number of cases. COR (95% CI) = crude odds ratio (95% confidence interval). AOR (95% CI) = adjusted odds ratio (95% confidence interval); adjusted for sex, age, smoking, secondhand smoke exposure, frequency of alcohol consumption, time spent at the company, past and present work with chemicals and prescription drug used in the past 12 months. ^1^ Adjusted for prior incident of head injury, coma, and concussion.

**Table 3:** Symptoms among warehouse and office workers at logistics companies.

Symptoms	Mean ^a^		COR	AOR
	Warehouse Workers (*n* = 122)	Office Workers (*n* = 136)	(95% CI)	(95% CI)
Weakness of the arms and feet ^1^	1.52	1.40	1.36 (0.82–2.23)	1.48 (0.64–3.44)
Decreased sensation in the arms and legs ^1^	1.18	1.21	0.99 (0.52–1.90)	0.74 (0.25–2.21)
Numbness or heaviness in the arms or legs ^1^	1.78	1.57	1.77 (1.11–2.83) *	3.99 (1.72–9.26) *
Trembling of hands ^1^	1.85	1.44	2.55 (1.57–4.13) *	1.52 (0.72–3.22)
Muscle cramps ^1^	1.43	1.42	0.92 (0.56–1.53)	2.00 (0.80–4.96)
Slurred speech ^1^	1.11	1.01	16.10 (2.07–125.01) *	11.86 (0.67–210.47)
Headache ^1^	2.08	2.62	0.35 (0.22–0.55) *	0.56 (0.28–1.13)
Dizziness ^1^	1.43	1.72	0.40 (0.25–0.66) *	0.85 (0.39–1.88)
Problems with balance, disturbed gait ^1^	1.12	1.72	0.61 (0.30–1.24)	0.72 (0.20–2.66)
An unpleasant taste in the mouth ^1^	1.45	1.17	2.97 (1.67–5.30) *	2.22 (0.90–5.49)
Nausea ^1^	1.40	1.57	0.57 (0.35–0.93) *	0.71 (0.32–1.56)
Feeling of general exhaustion, fatigue ^1^	1.95	1.96	0.95 (0.61–1.49)	0.69 (0.34–1.40)
Feeling irritable ^1^	1.66	1.79	0.65 (0.40–1.03)	0.63 (0.31–1.28)
Feeling depressed ^1^	1.37	1.66	0.37 (0.22–0.62) *	0.65 (0.28–1.49)
Rapid changes in mood ^1^	1.32	1.48	0.56 (0.32–0.97) *	1.11 (0.45–2.76)
Forgetfulness ^1^	1.52	1.74	0.51 (0.32–0.83) *	0.40 (0.18–0.87) *
Difficulty in concentrating ^1^	1.47	1.68	0.49 (0.30–0.79) *	0.56 (0.27–1.20)
Sleep disturbances ^1^	1.51	1.91	0.38 (0.23–0.62) *	0.36 (0.17–0.78) *
Feeling tired when you wake up ^1^	2.11	2.46	0.53 (0.34–0.83) *	0.40 (0.20–0.79) *
Stomach pain, cramps	1.35	2.14	0.14 (0.08–0.23) *	0.45 (0.21–1.00)
Diarrhea	1.88	1.85	1.01 (0.63–1.60)	0.91 (0.44–1.86)
Irritation of the eyes	1.60	1.99	0.54 (0.34–0.86) *	0.63 (0.30–1.33)
Dryness of throat and/or mouth	1.89	1.71	1.25 (0.79–1.99)	1.67 (0.79–3.54)
Throat irritation	1.75	1.65	1.17 (0.73–1.88)	1.76 (0.80–3.90)
A runny nose	1.84	1.94	0.79 (0.50–1.27)	1.60 (0.72–3.56)
Skin irritation	1.41	1.53	0.58 (0.34–0.99) *	1.18 (0.48–2.89)
Dry cough	2.20	1.93	1.42 (0.91–2.23)	2.32 (1.09–4.93) *
Wheezing in the chest	1.20	1.19	0.98 (0.49–1.96)	1.91 (0.45–8.18)
Shortness of breath	1.15	1.21	0.67 (0.33–1.38)	0.74 (0.20–2.68)
Chest tightness	1.25	1.22	0.98 (0.53–1.82)	1.13 (0.39–3.26)

^a^ Mean score of the experienced frequency of symptoms on a 5-item Likert scale (from 1 = Never to 5 = Very often). * Significant association (*p* < 0.05). COR (95% CI) = crude odds ratio (95% confidence interval). AOR (95% CI) = adjusted odds ratio (95% confidence interval); adjusted for sex, age, smoking, secondhand smoke exposure, frequency of alcohol consumption, time spent at the company, past and present work with chemicals, and prescription drug used in the past 12 months. ^1^ Adjusted for prior incident of head injury, coma, and concussion.

**Table 4:** Knowledge of and attitude to workplace chemical exposures among warehouse workers at logistics companies.

Knowledge of and Attitude to Workplace Chemical Exposures	Options	*N*	Proportion (*n* = 122)
How frequently do you work in closed spaces of transportation and storage?	Never	0	0%
	Rarely	2	1.64%
	Occasionally	8	6.56%
	Often	12	9.84%
	On every workday	97	79.51%
Have you ever heard about the chemical pollutants in closed spaces of transportation and storage?	Yes	46	37.70%
	No	76	62.30%
Where have you heard about the chemical pollutants? (*n* = 46)	Job training	11	9.02%
	Newspapers or television news	3	2.46%
	Workplace health and safety training	37	30.33%
	Boss	2	1.64%
	Co-worker	1	0.82%
	Other	1	0.82%
Which of the following best describes your general understanding of the risks of chemical pollutants in closed spaces?	I know a lot about chemical pollutants	12	9.84%
	I know a little about chemical pollutants	30	24.59%
	I do not know much about chemical pollutants	80	65.57%
How often do you think you work in closed spaces that can be polluted by chemicals?	Never	58	47.54%
	Rarely	26	21.31%
	Occasionally	16	13.11%
	Often	7	5.74%
	Always	15	12.30%
In your current job, how likely do you think you will be exposed to chemical pollutants in warehouses or closed transport devices (e.g., transport containers)?	Extremely unlikely	48	39.34%
	Unlikely	33	27.05%
	Neutral	20	16.39%
	Likely	10	8.20%
	Extremely likely	11	9.02%
How harmful do you think exposures to chemical pollutants in closed spaces could be to your health?	Not harmful	26	21.31%
	Not very harmful	31	25.41%
	Moderately harmful	33	27.05%
	Very harmful	22	18.03%
	Extremely harmful	10	8.20%

**Table 5:** Participation in specific workplace health and safety training related to chemical pollutants and the topics covered.

Workplace Preventive Measures	Options	*N*	Proportion (*n* = 122)
Have you completed any specific workplace health and safety training related to chemical pollutants in closed spaces of transportation and storage?	Yes	38	31.15%
	No	64	68.85%
What topics were covered in your specific workplace health and safety training? (*n* = 38, multiple choices allowed)	Identifying closed transport devices that may give off chemical fumes	23	18.85%
	Risks of exposure to chemical pollutants and/or how exposures occur	22	18.03%
	Properties of specific chemical pollutants—i.e., characteristic odors or other properties that may help identify if they are present, or responses to exposures such as skin and eye irritation, runny nose	14	11.48%
	Selection and use of personal protective equipment	34	27.87%
	Administrative controls—i.e., clearance procedures, exclusion zones during natural or mechanical ventilation periods, etc.	18	17.45%
	Reporting incidents	31	25.41%

**Table 6:** Association between completing specific workplace health and safety training related to chemical pollutants and the knowledge of and attitude to workplace chemical exposures in closed spaces of transportation and storage.

Knowledge of and Attitude to Workplace Chemical Exposures	Have You Completed Any Specific Workplace Health and Safety Training Related to Chemical Pollutants in Closed Spaces of Transportation and Storage? (Yes = 38; ^1^ No = 64)	
	COR	AOR
	(95% CI)	(95% CI)
Have you ever heard about the chemical pollutants in closed spaces of transportation and storage?	8.40 (3.53–19.99) *	7.85 (3.20–19.27) *
Which of the following best describes your general understanding of the risks of chemical pollutants in closed spaces?	9.06 (3.91–20.97) *	8.18 (3.47–19.27) *
How often do you think you work in closed spaces that can be polluted by chemicals?	2.95 (1.39–6.25) *	2.67 (1.24–5.78) *
In your current job, how likely do you think you will be exposed to chemical pollutants in warehouses or closed transport devices (e.g., transport containers)?	2.30 (1.11–4.80) *	1.88 (0.89–3.99)
How harmful do you think exposures to chemical pollutants in closed spaces could be to your health?	2.64 (1.30–5.38) *	2.21 (1.06–4.63) *

* Significant association (*p* < 0.05); ^1^ Reference value; COR (95% CI) = crude odds ratio (95% confidence interval); AOR (95% CI) = adjusted odds ratio (95% confidence interval); adjusted for sex, age, and time spent at the company.

**Table 7:** Workplace health and safety precautions applied by warehouse workers in closed spaces of transportation and storage.

		*N*	Proportion (*n* = 122)
When you are working in closed spaces of transportation and storage, what safety precautions do you or does your supervisor take? (multiple choices allowed)	Check documentation to see if the transport device (e.g., transport container) may give off chemical fumes	27	22.13%
	Open the transport device (e.g., transport container) taking reasonable care to avoid exposures to any chemical fumes or pollutants	29	23.77%
	Extract any chemical fumes or pollutants using a mechanical equipment (blower or extractor)	13	10.66%
	Extract any chemical fumes or pollutants using natural ventilation	39	31.97%
	Test the air in the transport device or in the warehouse using air testing equipment	6	4.92%
	Wear personal protective equipment	81	66.39%
	Other safety precautions	2	1.64%
	Do not take specific precautions	23	18.85%

**Table 8:** Association of completing specific workplace health and safety training related to chemical pollutants and being aware of chemical pollutants with applying preventive measures in closed spaces of transportation and storage at logistics companies.

Applied Workplace Preventive Measures	Have You Completed Any Specific Workplace Health and Safety Training Related to Chemical Pollutants in Closed Spaces of Transportation and Storage? (Yes = 38; ^1^ No = 64)		Have You Ever Heard about the Chemical Pollutants in Closed Spaces of Transportation and Storage? (Yes = 46; ^1^ No = 76)	
	COR	AOR	COR	AOR
	(95% CI)	(95% CI)	(95% CI)	(95% CI)
Check documentation to see if the transport device (e.g., transport container) may give off chemical fumes	5.99 (2.39–15.02) *	5.49 (2.13–14.14) *	3.15 (1.31–7.61) *	2.96 (1.18–7.44) *
Open the transport device (e.g., transport container) taking reasonable care to avoid exposures to any chemical fumes or pollutants	7.40 (2.96–18.51) *	7.16 (2.78–18.44) *	3.13 (1.32–7.38) *	3.04 (1.25–7.39) *
Extract any chemical fumes or pollutants using a mechanical equipment (blower or extractor)	9.64 (2.48–37.57) *	8.90 (1.96–40.27) *	2.99 (0.91–9.78)	2.96 (0.73–12.00)
Extract any chemical fumes or pollutants using natural ventilation	3.20 (1.42–7.19) *	2.48 (1.03–5.96) *	1.68 (0.77–3.66)	1.31 (0.55–3.10)
Test the air in the transport device or in the warehouse using air testing equipment	2.31 (0.45–12.03)	2.09 (0.37–11.70)	1.70 (0.33–8.79)	1.50 (0.26–8.54)
Wear personal protective equipment	0.96 (0.43–2.16)	0.98 (0.42–2.31)	0.92 (0.42–1.99)	1.05 (0.46–2.39)
Other safety precautions	2.24 (0.14–36.84)	1.27 (0.02–53.57)	1.67 (0.10–27.31)	5.97 (0.09–39.26)
Do not take specific precautions	0.56 (0.19–1.63)	0.66 (0.21–2.07)	0.39 (0.13–1.14)	0.36 (0.11–1.14)

* Significant association (*p* < 0.05). ^1^ Reference value. COR (95% CI) = crude odds ratio (95% confidence interval). AOR (95% CI) = adjusted odds ratio (95% confidence interval); adjusted for sex, age, and time spent at the company.

**Table 9:** Association between completing specific workplace health and safety training related to chemical pollutants and symptoms among warehouse workers at logistics companies.

Symptoms	Have You Completed Any Specific Workplace Health and Safety Training Related to Chemical Pollutants in Closed Spaces of Transportation and Storage? (Yes = 38; ^1^ No = 64)	
	COR	AOR
	(95% CI)	(95% CI)
Weakness of the arms and feet ^2^	1.05 (0.49–2.27)	1.55 (0.62–3.90)
Decreased sensation in the arms and legs ^2^	0.64 (0.22–1.89)	0.46 (0.12–1.76)
Numbness or heaviness in the arms or legs ^2^	1.41 (0.68–2.91)	1.44 (0.62–3.32)
Trembling of hands ^2^	0.99 (0.49–2.01)	1.44 (0.62–3.36)
Muscle cramps ^2^	1.13 (0.51–2.52)	1.35 (0.52–3.51)
Slurred speech ^2^	2.06 (0.64–6.62)	2.33 (0.54–10.01)
Headache ^2^	1.45 (0.72–2.95)	1.93 (0.86–4.35)
Dizziness ^2^	0.90 (0.41–1.99)	0.88 (0.33–2.34)
Problems with balance, disturbed gait ^2^	0.56 (0.15–2.15)	0.53 (0.10–2.88)
An unpleasant taste in the mouth ^2^	0.96 (0.44–2.09)	1.05 (0.41–2.69)
Nausea ^2^	0.62 (0.27–1.43)	0.46 (0.16–1.33)
Feeling of general exhaustion, fatigue ^2^	0.73 (0.36–1.50)	0.81 (0.35–1.89)
Feeling irritable ^2^	1.04 (0.50–2.15)	1.03 (0.45–2.36)
Feeling depressed ^2^	1.94 (0.84–4.48)	1.74 (0.66–4.55)
Rapid changes in mood ^2^	1.75 (0.72–4.21)	1.79 (0.65–4.91)
Forgetfulness ^2^	1.21 (0.57–2.56)	1.45 (0.62–3.42)
Difficulty in concentrating ^2^	1.05 (0.48–2.26)	1.17 (0.48–2.87)
Sleep disturbances ^2^	0.88 (0.39–1.97)	0.82 (0.30–2.20)
Feeling tired when you wake up ^2^	0.99 (0.50–1.97)	1.28 (0.58–2.83)
Stomach pain, cramps	0.59 (0.25–1.40)	0.48 (0.16–1.42)
Diarrhea	0.56 (0.27–1.15)	0.38 (0.16–0.91) *
Irritation of the eyes	0.78 (0.36–1.67)	0.86 (0.37–1.98)
Dryness of throat and/or mouth	0.31 (0.14–0.69) *	0.26 (0.10–0.64) *
Throat irritation	0.44 (0.21–0.95) *	0.33 (0.14–0.81) *
A runny nose	0.53 (0.24–1.17)	0.51 (0.20–1.30)
Skin irritation	0.69 (0.27–1.79)	0.80 (0.27–2.37)
Dry cough	0.62 (0.31–1.24)	0.43 (0.19–0.97) *
Wheezing in the chest	0.62 (0.19–2.04)	0.58 (0.12–2.67)
Shortness of breath	0.55 (0.14–2.09)	0.40 (0.07–2.33)
Chest tightness	0.92 (0.35–2.46)	0.50 (0.14–1.84)

* Significant association (*p* < 0.05); ^1^ Reference value; COR (95% CI) = crude odds ratio (95% confidence interval); AOR (95% CI) = adjusted odds ratio (95% confidence interval); adjusted for sex, age, smoking, secondhand smoke exposure, frequency of alcohol consumption, time spent at the company, past and present work with chemicals, and prescription drugs used in the past 12 months. ^2^ Also adjusted for prior incident of head injury, coma, and concussion.

## Data Availability

The data presented in this study are available on request from the authors (S.L. and B.Á.).
